# Conformation Determines the Seeding Potencies of Native and Recombinant Tau Aggregates

**DOI:** 10.1074/jbc.M114.589309

**Published:** 2014-11-18

**Authors:** Benjamin Falcon, Annalisa Cavallini, Rachel Angers, Sarah Glover, Tracey K. Murray, Luanda Barnham, Samuel Jackson, Michael J. O'Neill, Adrian M. Isaacs, Michael L. Hutton, Philip G. Szekeres, Michel Goedert, Suchira Bose

**Affiliations:** From the ‡ Medical Research Council Laboratory of Molecular Biology, Francis Crick Avenue, Cambridge CB2 0QH, United Kingdom,; § Eli Lilly and Co., Erl Wood Manor, Windlesham, Surrey GU20 6PH, United Kingdom, and; the ¶ UCL Institute of Neurology, Queen Square, London WC1N 3BG, United Kingdom

**Keywords:** Aggregation, Neurodegenerative Disease, Prion, Protein Conformation, Tau Protein, Tauopathy, Seed-competent Aggregation

## Abstract

Intracellular Tau inclusions are a pathological hallmark of several neurodegenerative diseases, collectively known as the tauopathies. They include Alzheimer disease, tangle-only dementia, Pick disease, argyrophilic grain disease, chronic traumatic encephalopathy, progressive supranuclear palsy, and corticobasal degeneration. Tau pathology appears to spread through intercellular propagation, requiring the formation of assembled “prion-like” species. Several cell and animal models have been described that recapitulate aspects of this phenomenon. However, the molecular characteristics of seed-competent Tau remain unclear. Here, we have used a cell model to understand the relationships between Tau structure/phosphorylation and seeding by aggregated Tau species from the brains of mice transgenic for human mutant P301S Tau and full-length aggregated recombinant P301S Tau. Deletion of motifs ^275^VQIINK^280^ and ^306^VQIVYK^311^ abolished the seeding activity of recombinant full-length Tau, suggesting that its aggregation was necessary for seeding. We describe conformational differences between native and synthetic Tau aggregates that may account for the higher seeding activity of native assembled Tau. When added to aggregated Tau seeds from the brains of mice transgenic for P301S Tau, soluble recombinant Tau aggregated and acquired the molecular properties of aggregated Tau from transgenic mouse brain. We show that seeding is conferred by aggregated Tau that enters cells through macropinocytosis and seeds the assembly of endogenous Tau into filaments.

## Teaser

**Background:** Characteristics of seed-competent Tau are unknown.

**Results:** Native Tau aggregates have a higher seeding potency than recombinant Tau aggregates. Recombinant Tau acquires the conformation and potency of native Tau aggregates by seeded assembly.

**Conclusion:** Conformation determines the seeding potencies of Tau aggregates.

**Significance:** Understanding the properties of seed-competent Tau gives insight into disease mechanisms.

## Introduction

Soluble microtubule-associated protein Tau assembles into insoluble, filamentous, and hyperphosphorylated intracellular inclusions in a number of human neurodegenerative diseases, which include Alzheimer disease (AD),^8^ tangle-only dementia, Pick disease, argyrophilic grain disease (AGD), progressive supranuclear palsy, corticobasal degeneration, and chronic traumatic encephalopathy (1). In the adult human brain, six Tau isoforms are generated from *MAPT*, the Tau gene, through alternative mRNA splicing (2). Alternative splicing of exon 10 gives rise to three isoforms with three microtubule-binding repeats each (3R) and three isoforms with four microtubule-binding repeats each (4R). The repeats are 31 or 32 amino acids in length and are located toward the carboxyl terminus. In addition, the presence of inserts of 29 or 58 amino acids or no insert in the amino terminus gives rise to 1N, 2N, or 0N forms of each 3R and 4R Tau. Full-length Tau assembles through the repeats that form the core of the filament, with the amino-terminal half and the carboxyl terminus forming the “fuzzy coat” of the filament (3, 4). The fuzzy coat surrounds the filament core and resembles a two-layered polyectrolyte brush, which is made of the unstructured long amino-terminal and short carboxyl-terminal domains of Tau (5). The isoform composition of Tau filaments can vary between diseases. Thus, in AD, tangle-only dementia, and chronic traumatic encephalopathy, both 3R and 4R Tau make up the neurofibrillary lesions (6–8), whereas in Pick disease, 3R Tau predominates in the neuronal inclusions (9). The assembly of 4R Tau into filaments is characteristic of progressive supranuclear palsy, corticobasal degeneration, and AGD (10–13).

Mutations in *MAPT* cause familial forms of frontotemporal dementia, establishing that Tau protein dysfunction is sufficient to cause neurodegeneration and dementia (14–16). These mutations cause the formation of inclusions made of full-length hyperphosphorylated filamentous Tau; depending on the nature of the *MAPT* mutation, the inclusions contain all six Tau isoforms, or predominantly 4R Tau (17).

Filaments can be assembled from non-phosphorylated full-length recombinant Tau in the presence of sulfated glycosaminoglycans, RNA, or free fatty acids (18–21). Heparin compacts the repeats and induces the dimerization of Tau, with filaments growing through monomer addition (22, 23). Sequences in the second (amino acids 275–280, VQIINK) and third (amino acids 306–311, VQIVYK) repeats are essential for the heparin-induced assembly of Tau into filaments (24, 25). Tau assembly involves the transition from a natively unfolded monomer to a structured filament with increased β-sheet content.

In AD, misfolded, hyperphosphorylated Tau first accumulates in the locus coeruleus, from where it appears to spread to the entorhinal cortex, hippocampus, and neocortex. This differential distribution underlies the Braak stages of Tau pathology (26, 27). Stereotypical temporospatial spreading of Tau pathology has also been described in AGD (28). Transmission and spreading of Tau pathology can be shown experimentally (29). Thus, brain extracts from mice transgenic for human mutant P301S Tau with abundant silver-positive Tau inclusions, when injected into the brains of ALZ17 mice expressing human wild-type Tau (lacking Tau inclusions), induced the slow assembly of wild-type Tau into silver-positive inclusions. Moreover, this Tau pathology was observed to spread to neighboring brain regions (30). Aggregated recombinant Tau was also sufficient to convert soluble Tau into aggregates (31, 32). Infusion of P301S Tau extracts from the hindbrain of symptomatic mice into the hippocampus and overlying cerebral cortex of non-symptomatic mice transgenic for human P301S Tau induced the formation of Tau inclusions in the hippocampus, which spread rapidly (2–4 weeks) to synaptically connected brain regions (33). This is reminiscent of the prion protein, for which incubation times and infectivity depend on the correspondence between the conformation of the infectious prion and that of the host prion protein, as determined principally by their similarities in amino acid sequence (34). The induction and spreading of Tau pathology has also been demonstrated following the restricted expression of human mutant Tau (35, 36). A recent study has shown that the injection of brain homogenates from human tauopathies into the brains of mice transgenic for human wild-type Tau induced the formation of silver-positive Tau inclusions (37). Some inclusions also formed following the injection of the same human brain homogenates into wild-type mice. This work revealed the likely existence of distinct conformers (or strains) of assembled 4R Tau, because following the intracerebral injection of the corresponding brain extracts, the light microscopic hallmark lesions of AGD, progressive supranuclear palsy, and corticobasal degeneration were recapitulated. Prion-infected tissues harbor multiple conformers of the prion protein (38), and prions can evolve in response to environmental pressures (39, 40). It remains to be determined whether this also holds true for distinct Tau conformers.

The intercellular transfer of Tau inclusions has been shown in cultured cells (41–45). Aggregates made of recombinant Tau or Tau filaments extracted from the brains of AD patients were taken up by cells and induced the aggregation of cytoplasmic Tau. The internalization of aggregated Tau depended on the presence of sulfated glycosaminoglycans at the cell surface (46). Following uptake and nucleation, aggregated Tau was released into the extracellular space (47). The mechanism by which Tau pathology spreads is not well understood. A better understanding of the role of Tau conformation in propagation may open new therapeutic avenues for AD and other tauopathies.

Here we have developed a cell model to understand the relationship between Tau structure/phosphorylation and seeding. Using either non-phosphorylated synthetic Tau aggregates (made from recombinant Tau) or hyperphosphorylated native Tau aggregates (from the brains of mice transgenic for human mutant P301S Tau), we show that pathological Tau was capable of entering cells, where it triggered the aggregation of soluble Tau in an aggregation-dependent manner. Sarkosyl-insoluble Tau from the brains of mice transgenic for human mutant P301S Tau was more effective at seeding than Sarkosyl-insoluble aggregates of unphosphorylated and hyperphosphorylated recombinant Tau. Using proteinase K digestion and guanidine hydrochloride disaggregation, we identified conformational differences that may account for the differing seeding activities of these preparations. The addition of soluble recombinant Tau to aggregated Tau seeds from the brains of mice transgenic for P301S Tau led to its assembly, with a seeding potency and resistance to disaggregation with guanidine hydrochloride like that of Sarkosyl-insoluble Tau from the transgenic mouse brain.

## EXPERIMENTAL PROCEDURES

### Preparation of Tau Seeds

#### 

##### Preparation of Mouse Brain Homogenates

Homozygous mice transgenic for human 0N4R Tau with the P301S mutation (TgP301S Tau) with a severe paraparesis (48) were killed by cervical dislocation followed by exsanguination. Brains were dissected and stored at −80 °C. Whole brains were homogenized at 10% (w/v) in sterile phosphate-buffered saline (PBS), and their protein content was determined using bicinchoninic acid (BCA).

##### Purification of Recombinant Tau

Human P301S 0N4R Tau was expressed as described (49). Bacteria were harvested following a 10-min centrifugation at 5,000 × *g*. Cleared lysates were prepared in 25 mm Tris-HCl, pH 7.4, 10 mm EDTA, 0.1 mm dithiothreitol (DTT), and 0.1 mm phenylmethanesulfonyl fluoride (PMSF) using a cell disruptor (at a pressure of 25,000 p.s.i.), followed by centrifugation at 18,000 × *g* for 20 min. The lysate was passed through a DE52 anion exchange column, followed by a phosphocellulose cation exchange column. Tau fractions were eluted with 500 mm NaCl, precipitated using 25% ammonium sulfate, and run on a Superdex 200 HiLoad 16/60 column. The resulting fractions with the highest Tau content were passed through a Mono S 50/50 GL cation exchange column and eluted with a 50–275 mm NaCl gradient. Fractions were then pooled and dialyzed against 40 mm HEPES, pH 7.4, containing 0.1 mm DTT. Aliquots of recombinant Tau were snap-frozen and stored at −20 °C. To obtain monomeric Tau, purified recombinant Tau was centrifuged at 100,000 × *g* at 4 °C for 1 h, and the supernatant was used.

##### In Vitro Phosphorylation of Recombinant Tau

Recombinant P301S Tau was phosphorylated as described previously (50). Two μm Tau was incubated for 6 h at 30 °C with 1 unit/ml cAMP-dependent protein kinase (PKA) (New England Biolabs) in 25 mm Tris-HCl, pH 7.4, 0.1 mm EGTA, 2 mm 4-(2-aminoethyl) benzenesulfonyl fluoride hydrochloride, 10 mm magnesium acetate, 2 mm ATP, and 50 μg/ml heparin (Sigma). After 6 h, 1 unit/ml stress-activated protein kinase 4 (SAPK4) (Millipore) was added, and the reaction was allowed to proceed for an additional 6 h. The samples were then incubated for 5 min at 95 °C, before centrifugation for 15 min at 100,000 × *g*. The supernatants were used.

##### Heparin-induced Assembly of Recombinant Tau

Recombinant Tau (60 μm) was assembled by incubation with 400 μg/ml heparin (Sigma) in 30 mm MOPS, pH 7.2, at 37 °C for 3 days, as described (18).

##### In Vitro Seeded Assembly of Recombinant Tau

Recombinant P301S Tau was diluted to 135 μg/ml in PBS plus 1 mm DTT and incubated at 37 °C in an 800-μl reaction volume with or without 5% (v/v) TgP301S Tau Sarkosyl-insoluble brain extract. An aliquot of the samples was added to 15 μm thioflavin T in a 384-well plate, and thioflavin T fluorescence was monitored over time with excitation and emission filters set to 444 and 485 nm, respectively, in an Envision (PerkinElmer Life Sciences) plate reader. Fluorescence readings were taken every 20 min, with agitation in between each reading. The aggregation reaction was stopped, by snap-freezing the samples, when the thioflavin T signal reached a plateau. Samples were Sarkosyl-extracted, as described below, and the pellets were washed and resuspended in 50 mm Tris-HCl, pH 7.4. Samples were run on SDS-PAGE and probed with total Tau antibody DA9. Equal amounts of Tau protein were then added to HEK Trex P301S 1N4R Tau-inducible cells following the seeding paradigm described below.

##### Preparation of Sarkosyl-insoluble Tau

Sarkosyl-insoluble Tau was prepared by diluting 5 mg of total protein of brain homogenate in 1 ml of A68 buffer (10 mm Tris-HCl, pH 7.4, 0.8 m NaCl, 1 mm EGTA, 5 mm EDTA, 10% sucrose), supplemented with protease and phosphatase inhibitors, followed by centrifugation at 13,000 × *g* at 4 °C for 20 min. The supernatants were retained and kept on ice, and the pellets were resuspended in 500 μl of A68 buffer and centrifuged for a further 20 min. Both supernatants were combined. Assembled recombinant Tau was diluted directly in A68 buffer. Samples were then incubated with 1% sodium lauroyl sarcosinate (Sarkosyl, Sigma) at room temperature for 1 h in a flat rotating shaker at 700 rpm followed by centrifugation at 100,000 × *g* at 4 °C for 1 h. The pellets were washed and resuspended in 50 mm Tris-HCl, pH 7.4. Samples were used immediately or snap-frozen in liquid nitrogen and stored at −80 °C.

##### Immunopurification of TgP301S Tau

Tau was immunopurified from the brains of symptomatic TgP301S Tau mice (aged 4.5 months) as described (52) using an MC1 affinity column. By ELISA, the relative levels of AT8- and MC1-positive Tau were similar to those obtained following Sarkosyl extraction (data not shown).

### Antibodies

Phosphorylation-independent anti-Tau antibodies BR133 (amino terminus), BR304 (first amino-terminal insert), BR135 (repeat region), and BR134 (carboxyl terminus) have been described (2).

The following antibodies were kind gifts from Peter Davies (Albert Einstein College of Medicine, New York): total Tau, DA9 (amino acids 102–140) (53) and TG5 (amino acids 220–240) (54); conformationally changed Tau, MC1 (55); phosphorylated Tau, MC6 (Ser(P)^235^) (56) and PG5 (Ser(P)^409^) (52).

The phosphorylation-dependent anti-Tau antibodies AT8 (Ser(P)^202^/Thr(P)^205^) (57) and AT100 (Ser(P)^212^/Thr(P)^214^/Thr(P)^217^) (50) as well as the phosphorylation-independent antibody HT7 (amino acids 159–163), were purchased from Thermo (Pierce); antibody Ser(P)^422^ was purchased from Abgent, the 1N Tau antibody was purchased from Covance, and the GAPDH antibody was purchased from Millipore.

### Cell Culture Tau Seeding Assay

HEK293T cells (ECACC) were transiently transfected with pcDNA3.1 constructs encoding human Tau using Lipofectamine 2000 (Invitrogen). The cells were split the following day and grown overnight. They were then seeded with Sarkosyl-insoluble Tau (at the concentrations stated in the figure legends) diluted in OptiMEM (Invitrogen). After 3 h, the seed/OptiMEM mix was removed and replaced with fresh complete medium (DMEM plus 10% fetal calf serum (FCS)). Cells were then incubated for 3 days.

For the production of an inducible cell line, HEK-TREx-293 cells (Invitrogen) were transfected with human 1N4R P301S Tau/pcDNA 4T/O using FuGene 6 (Roche Applied Science). Clones were selected in the presence of 5 μg/ml blasticidin and 200 μg/ml zeocin. Tau expression was induced in medium containing 1 μg/ml tetracycline for 24–48 h, and expression levels were evaluated by Western blotting with anti-Tau antibody DA9.

### Western Blot Analysis

Cell extracts were prepared in A68 buffer supplemented with protease and phosphatase inhibitors by lysis on ice for 30 min and a freeze-thaw cycle on dry ice. The samples were normalized to that with the lowest protein content, as determined by BCA. Total lysate samples were retained, and the remaining extracts were incubated with or without 1% Sarkosyl at room temperature for 1 h in a flat rotating shaker at 700 rpm. Samples were then centrifuged at 100,000 × *g* for 1 h at 4 °C, and the pellets were washed and resuspended in 15 μl of 50 mm Tris-HCl, pH 7.4. An aliquot of the supernatant was retained as the soluble fraction. The samples were resolved using 4–20% Tris-glycine gels. Tau protein was detected by enhanced chemiluminescence (ECL). The primary antibodies used were BR134, BR304, BR133, BR135 (all at 1:5,000), AT8 (1:1,000), DA9 (1:2,000), and HT7 (1:2,500). Anti-GAPDH (1:3,000) was used as the loading control.

### AlphaScreen Assays

The level of total and phosphorylated Tau were quantitated by AlphaScreen assays. The AlphaScreen technology is a sensitive and quantitative assay (similar to a sandwich ELISA) for the detection of molecules of interest in biological fluids (58). A biotinylated anti-analyte antibody binds to streptavidin donor beads while a second anti-analyte antibody is conjugated to acceptor beads. In the presence of the analyte, the beads come into close proximity. The excitation of the donor beads provokes the release of singlet oxygen molecules that triggers energy transfer in the acceptor beads, resulting in light emission. AlphaScreen assays are potentially sensitive to the aggregation state of the analyte; therefore, this method cannot quantitate soluble *versus* insoluble analytes in absolute terms.

AlphaScreen assays (PerkinElmer Life Sciences) were carried out according to the manufacturer's instructions using specific anti-Tau antibodies. They used either biotinylated DA9 or biotinylated AT8 bound to donor beads and TG5 conjugated to acceptor beads (Ab-ACC). Ten μl/well of antibody mix consisting of Ab-ACC and biotinylated antibody were added to 384-well assay plates (Greiner), together with 5 μl/well of sample or standard diluted in AlphaScreen assay buffer (0.1% casein in Dulbecco's phosphate-buffered saline). The plates were incubated overnight in the dark at 4 °C. Five μl of streptavidin-coated donor beads diluted in AlphaScreen buffer were then added to each well, and the plates were incubated in the dark with gentle agitation at room temperature for 4 h. They were read at excitation 680 nm and emission 520–620 nm using an Envision plate reader (PerkinElmer Life Sciences). The AlphaScreen counts or total Tau levels, relative to a standard of paired helical filaments purified from AD brain per μg of protein or per ml of sample, were calculated.

### Fluorescence Imaging

#### 

##### Immunostaining

Cells were grown on poly*-*l-lysine-coated coverslips, fixed with 4% paraformaldehyde for 20 min, and probed by immunostaining. The antibodies used were AT100 (1:1,000) or HT7 (1:500) with goat anti-mouse IgG secondary antibody conjugated to Alexa Fluor 488; PG5 (1:1,000) with anti-IgG3 conjugated to Alexa Fluor 488; and 1N Tau (1:100) with anti-IgG1 conjugated to Alexa Fluor 568. Washings were carried out with PBS and antibody solutions prepared with either 0.1% Triton X-100 plus 1% bovine serum albumin (BSA) in PBS or 5% milk in PBS. DAPI or Hoechst staining was used to visualize nuclei. Confocal images were acquired using an Olympus Fluoview 1000 or Zeiss 710, with a 60× oil objective. High-content imaging was performed using a BD Pathway system (BD Biosciences).

##### Thioflavin S Staining

To reduce background staining, cells were incubated with 0.3% potassium permanganate for 5 min, washed, and incubated with 1% sodium metabisulfite plus 1% oxalic acid for 20–40 s (33). Cells were then stained with 0.05% Thioflavin S (Sigma) in 50% ethanol in the dark for 8 min, differentiated in 80% ethanol, and washed three times in water.

##### Perturbation of Tau Uptake into Cells

The uptake of TgP301S and synthetic P301S Tau seeds by cells was compared. The concentrations of both seeds were normalized by Western blotting before use. The cells were incubated with either 100 μm ethylisopropyl amiloride (EIPA) (Sigma) or 300 nm latrunculin A (Sigma) for 30 min prior to and during the 3-h exposure to Tau seeds. For co-localization with dextran, cells were incubated for 1 h with 50 μg/ml dextran conjugated to Alexa Fluor 594 (*M*_r_ 10,000; Invitrogen), plus 500 nm TgP301S Tau or synthetic Tau aggregates diluted in OptiMEM (Invitrogen). Cells were then washed with PBS and incubated with 0.0125% trypsin plus EDTA (Invitrogen) for 3 min at room temperature to remove extracellular Tau. Immunostaining with HT7 was as described.

### Proteinase K Digestion

Tau seeds were digested with 0.25–10 μg/ml proteinase K in PBS for 30 min at 21 °C. Digestion was stopped with 5 mm PMSF, and SDS-loading buffer was added. The samples were resolved on 4–12% bis-tris gels with MES running buffer, and Tau protein was detected by ECL. The primary antibody used was BR135 (1:5,000).

### Guanidine Hydrochloride Disaggregation

Tau seeds were disaggregated with 1–6 m guanidine hydrochloride in 50 mm Tris HCl, pH 8, for 1 h at 21 °C. The samples were then centrifuged at 100,000 × *g* for 1 h at 4 °C. The pellets were washed and resuspended in 50 mm Tris-HCl, pH 7.4, and SDS-loading buffer was added. The samples were resolved on 4–20% Tris-glycine gels, and Tau protein was detected by ECL. The primary antibody used was HT7 (1:2,500).

### Electron Microscopy

Tau seeds were placed on 400 meshed Formvar/carbon film-coated copper grids (Sigma) for 3 min, blocked with PBS plus 0.1% gelatin for 10 min, and incubated with anti-Tau antibody HT7 (1:50) for 1 h. Grids were then washed and incubated with secondary antibody 10-nm gold conjugate (1:20; Sigma) for 1 h before being washed and stained with uranyl acetate. Images were taken on a Philips Spirit transmission electron microscope.

### Flow Cytometry

Uptake of monomeric, aggregated full-length recombinant Tau or Tau immunopurified from TgP301S mice was measured by flow cytometry. Tau was labeled with DyLight 488 NHS-ester according to the manufacturer's guidelines (Thermo). Cells were exposed to 500 nm monomeric or aggregated recombinant Tau (assuming complete assembly) diluted in OptiMEM for 5–120 min prior to washing with cold PBS and detachment with trypsin/EDTA. This step also served to digest all extracellular Tau. Cells were resuspended in cold PBS and analyzed immediately (Sony Eclipse). Tau uptake was blocked by incubation with either 100 μm EIPA or 300 nm latrunculin A for 30 min prior to and during the 30-min exposure to Tau.

## RESULTS

### 

#### 

##### Sarkosyl-insoluble Tau from the Brains of Mice Transgenic for Human P301S Tau Induces the Aggregation of Soluble Human P301S Tau in HEK293T Cells

**FIGURE 1.**
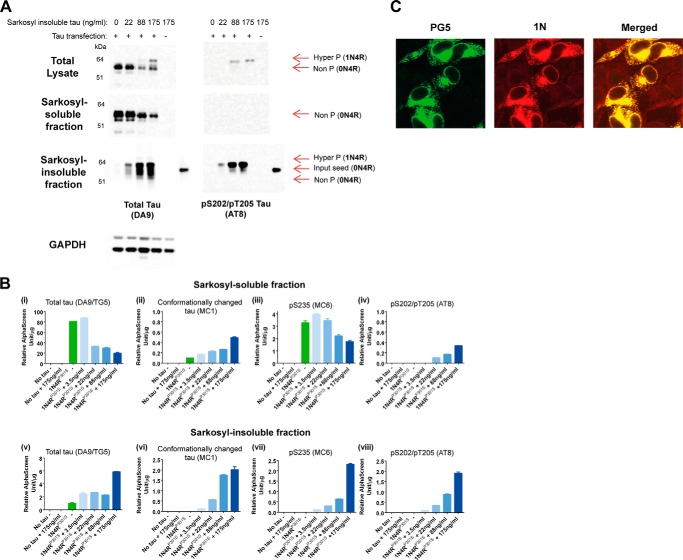
*A*, Western blot with anti-Tau antibodies DA9 (phosphorylation-independent) and AT8 (Ser(P)^202^/Thr(P)^205^) of the total lysate and Sarkosyl-soluble and -insoluble fractions from HEK293T cells transiently transfected with or without P301S 1N4R Tau DNA. The cells were exposed for 3 h to increasing amounts of Sarkosyl-insoluble material from the brains of symptomatic TgP301S 0N4R Tau mice, followed by 3 days of incubation. A total lysate loading control for GAPDH is also shown. *B*, AlphaScreen showing levels of total Tau (DA9) (*i* and *v*), conformationally changed Tau (MC1) (*ii* and *vi*), and Tau phosphorylated at Ser^235^ (MC6) (*iii* and *vii*) or Ser^202^/Thr^205^ (AT8) (*iv* and *viii*) in HEK 293T cells transiently transfected with or without human P301S 1N4R Tau, exposed for 3 h to increasing amounts of Sarkosyl-insoluble material from the brains of symptomatic TgP301S 0N4R Tau mice, followed by 3 days of incubation. The results are the means ± S.D. (*n* = 3). Absolute quantification of the levels of Sarkosyl-soluble (*top*) and Sarkosyl-insoluble (*bottom*) Tau cannot be made because the magnitude of the signal is dependent on the aggregation state of Tau. *C*, PG5 (Ser(P)^409^)-positive Tau inclusions (*green*) in inducible HEK 293T cells expressing human P301S 1N4R Tau, following exposure to Sarkosyl-insoluble material from the brains of symptomatic TgP301S 0N4R Tau mice for 3 h followed by 3 days of incubation. Total Tau was visualized with an antibody specific for 1N Tau (phosphorylation-independent) (*red*).

We examined the ability of Sarkosyl-insoluble Tau extracted from the brains of TgP301S Tau mice with a severe paraparesis to seed the assembly of Tau in HEK293T cells transiently transfected with P301S 1N4R Tau. Cells expressing Tau were exposed to Tau seeds for 3 h, washed, and then grown for a further 3 days. Expressed Tau was recruited into the Sarkosyl-insoluble fraction, as detected by antibodies DA9 (phosphorylation-independent) and AT8 (Ser(P)^202^/Thr(P)^205^) (Fig. 1 *A*, *bottom panel* of Tau blots). A dose-dependent increase in Sarkosyl-insoluble, AT8-positive 1N4R Tau was observed with increasing 0N4R seed concentrations, which was accompanied by a dose-dependent decrease in AT8-negative, Sarkosyl-soluble Tau (Fig. 1*A*, compare *middle* and *bottom panels* of Tau blots). The use of two different isoforms (0N4R Tau seeds and expressed 1N4R Tau), in conjunction with specific antibodies, allowed the induced Tau aggregates to be distinguished from those added to the cells (Fig. 1, *A* (*bottom panel* of Tau blots) and *C*). No inclusions were observed in HEK293 cells that were exposed to Tau seeds but did not express Tau.

Seeding did not differ between 0N4R and 1N4R expressed Tau and was more efficient when P301S Tau rather than wild-type Tau was expressed (data not shown). Cells expressing P301S 1N4R Tau were used in all subsequent experiments unless otherwise stated.

An AlphaScreen was used to measure the levels of different Tau species (Fig. 1*B*). Sarkosyl-soluble Tau was phosphorylated at the MC6 (Ser(P)^235^) epitope. Following treatment with TgP301S Tau seed, there was a dose-dependent reduction in Sarkosyl-soluble total Tau (Fig. 1*B* (*i*)) and Tau phosphorylated at the MC6 (Ser(P)^235^) epitope (Fig. 1*B* (*iii*)) and a parallel increase in Sarkosyl-insoluble total (Fig. 1*B* (*v*)) and MC6 (Ser(P)^235^)-positive Tau (Fig. 1*B* (*vii*), suggesting that soluble Tau was being converted into insoluble Tau aggregates, consistent with the findings in Fig. 1*A*. In the Sarkosyl-soluble fraction, only a small amount of Tau was MC1 (conformationally changed Tau)-positive (0.1 AlphaScreen units/μg); however, this species increased to 0.49 AlphaScreen units/μg (at the highest seed concentration) following seed treatment for 3 h and growth for 3 days (Fig. 1*B* (*ii*)). In addition, this fraction was also AT8-positive following seed treatment but not upon soluble Tau expression and no seeding (from 0 in unseeded cells to 0.33 AlphaScreen units/μg at the highest seed concentration; Fig. 1*B* (*iv*)). This could reflect an initial change in Tau conformation and subsequent phosphorylation upon seeding, followed by Tau becoming insoluble, as the concentration of these species increased, or the establishment of equilibria, whereby small misfolded and hyperphosphorylated soluble Tau species formed as a consequence of secondary nucleation events at the ends of filaments. We cannot completely exclude the possibility of an incomplete separation between Sarkosyl-soluble and Sarkosyl-insoluble material (although no differences in the amounts of MC1 (conformationally changed)- and AT8 (Ser(P)^202^/Thr(P)^205^)-positive Tau in the Sarkosyl-soluble fractions were observed following 100,000 × *g* centrifugations at 30 min, 1 h, and 24 h). Following incubation with Tau seeds, a dose-dependent increase in the levels of MC1 (conformationally changed)- and AT8 (Ser(P)^202^/Thr(P)^205^)-positive Sarkosyl-insoluble Tau was observed (Fig. 1*B* (*vi* and *viii*).

An inducible HEK293T cell line stably expressing P301S 1N4R Tau was used to further investigate seeded assembly. Cells were exposed to the Sarkosyl-insoluble fraction from the brains of TgP301S Tau mice 24 h after the induction of Tau expression and treated with 0.0125% trypsin to remove extracellular Tau. Exposure to 0.0125% trypsin/EDTA for 3 min at 21 °C completely removed extracellular, surface-bound Tau aggregates (data not shown). Tau aggregates were examined by fluorescence confocal microscopy. In cells expressing P301S 1N4R Tau, seeding gave rise to 1N- and PG5 (Ser(P)^409^)-positive Tau (Fig. 1*C*).

**FIGURE 2.**
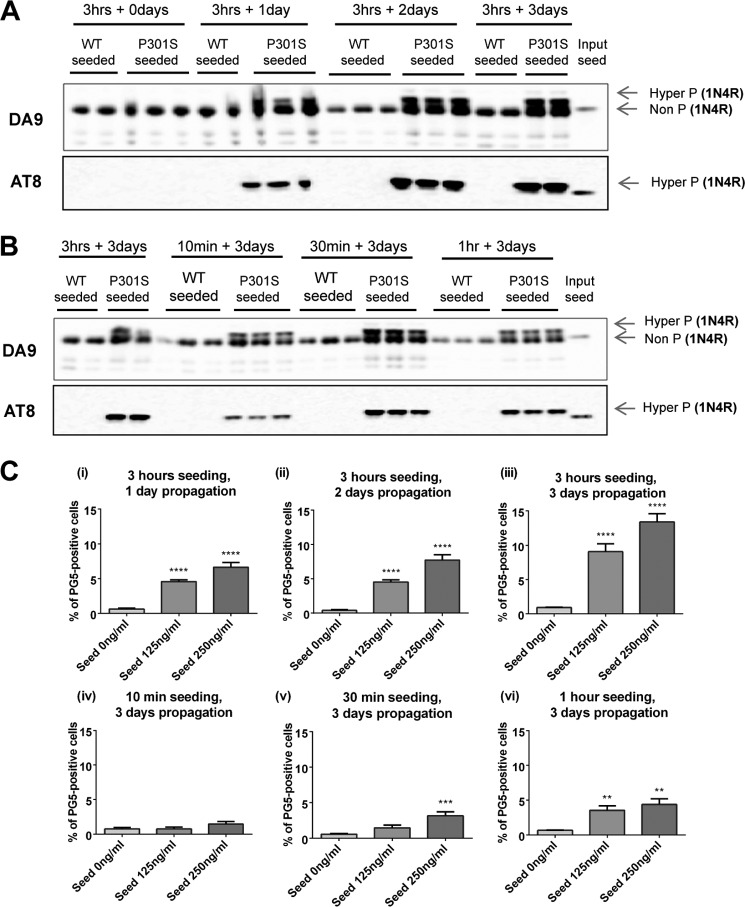
*A* and *B*, time course of propagation (*A*) and seeding (*B*) following inoculation of inducible HEK293T cells expressing human P301S 1N4R Tau with the Sarkosyl-insoluble fraction from the brains of non-transgenic (*wt*) or TgP301S Tau mice. The samples were fractionated by centrifugation of total lysate at 100,000 × *g*, and the insoluble fraction was analyzed by Western blotting using anti-Tau antibodies DA9 (phosphorylation-independent) and AT8 (Ser(P)^202^/Thr(P)^205^). Representative blots from three separate experiments are shown. *C*, PG5 (Ser(P)^409^)-positive Tau inclusions in cells, as measured using high-content imaging, confirmed the time dependence of propagation (*i–iii*) and seeding (*iv–vi*). The results are the means ± S.D. (*error bars*) (*n* = 6); **, *p* < 0.01; ***, *p* < 0.001; ****, *p* < 0.0001 *versus* unseeded (analysis of variance).

Following seed addition, we observed a time-dependent increase in AT8 (Ser(P)^202^/Thr(P)^205^)-positive insoluble Tau, which was absent from cells seeded with Sarkosyl-insoluble material from non-transgenic mouse brain (Fig. 2*A*). We also examined the effect of varying the incubation times with seeds from 10 min to 3 h, followed by trypsin treatment and further growth for 3 days. Ten min of incubation was sufficient to seed the formation of AT8 (Ser(P)^202^/Thr(P)^205^)-positive insoluble Tau. Increasing the incubation time resulted in the formation of more insoluble Tau (Fig. 2*B*). These findings were confirmed by quantitative high-content imaging using antibody PG5 (Ser(P)^409^) to visualize the Tau inclusions (Fig. 2*C*).

##### Sarkosyl Treatment Enriches for Seed-competent Tau

**FIGURE 3.**
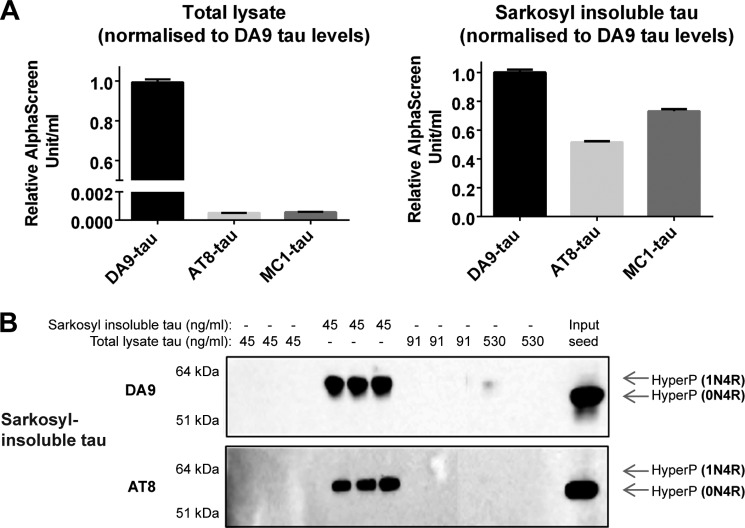
*A*, AlphaScreen assays showing the levels of DA9 (phosphorylation-independent)-, MC1 (conformationally changed)-, and AT8 (Ser(P)^202^/Thr(P)^205^)-positive Tau in the total lysate and the Sarkosyl-insoluble fraction from the brains of symptomatic TgP301S Tau mice. The results are the means ± S.D. (*error bars*) (*n* = 3). The levels of AT8- and MC1-positive Tau are expressed relative to those of DA9, taken as 1.0. *B*, Western blot showing DA9- and AT8-positive Tau in the Sarkosyl-insoluble fraction of HEK 293T cells expressing P301S 1N4R Tau, following the addition of either brain lysate or Sarkosyl-insoluble material (normalized for DA9-positive Tau) for 3 h, followed by 3 days of incubation.

The Sarkosyl-insoluble fraction from the brains of TgP301S Tau mice was compared with total brain lysate. Tau levels were normalized, and the levels of MC1 (conformationally changed)- and AT8 (Ser(P)^202^/Thr(P)^205^)-positive Tau determined by AlphaScreen assays. Sarkosyl treatment enriched for MC1 (conformationally changed)- and AT8 (Ser(P)^202^/Thr(P)^205^)-positive Tau relative to the total brain lysate (∼130- and ∼100-fold, respectively, taking into account that the Sarkosyl-insoluble pellet was resuspended in one-tenth of the volume of the total lysate) (Fig. 3*A*). We next compared the seeding abilities of these preparations. Whereas Sarkosyl-insoluble TgP301S Tau led to the accumulation of Sarkosyl-insoluble AT8 (Ser(P)^202^/Thr(P)^205^)-positive Tau, the total brain lysate failed to seed at this concentration (Fig. 3*B*).

##### Monomeric Tau Is Internalized by Cells but Fails to Seed

**FIGURE 4.**
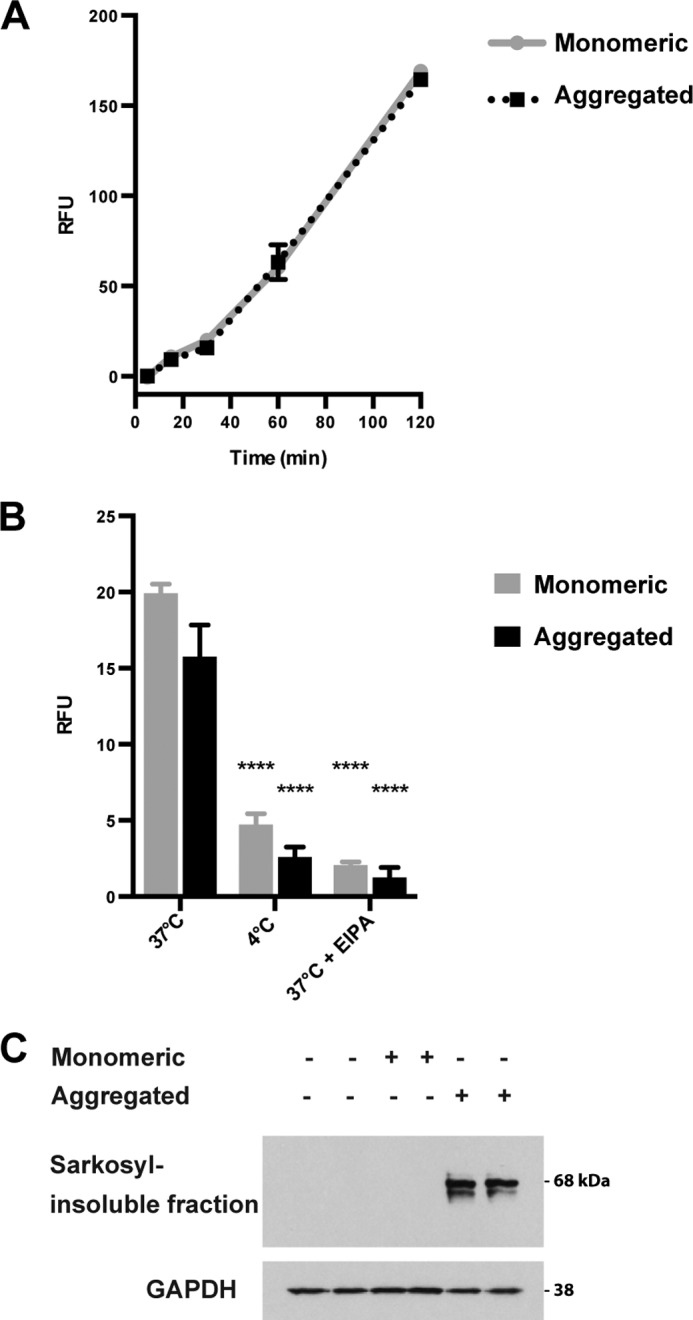
*A*, cellular uptake of 500 nm fluorescently labeled monomeric recombinant P301S Tau (*gray line*) and 500 nm fluorescently labeled aggregated recombinant P301S Tau (*black line*) over 120 min as measured by flow cytometry. Uptake was not significantly different between the two groups. The results are the means ± S.D. (*error bars*) (*n* = 3). *B*, uptake of 500 nm fluorescently labeled monomeric (*gray bars*) or aggregated (*black bars*) P301S Tau after 30 min measured by flow cytometry. Incubation at 4 °C or with 100 μm EIPA significantly inhibited the uptake of both monomeric and aggregated Tau. The results are the means ± S.D. (*n* = 3); ****, *p* < 0.0001 *versus* controls (analysis of variance); 10,000 cells/well were analyzed by flow cytometry. *RFU*, relative fluorescence units. *C*, Western blot with anti-Tau antibody HT7 (phosphorylation-independent) of the Sarkosyl-insoluble fraction from HEK 293T cells expressing P301S 1N4R Tau, unseeded or seeded for 3 h with 500 nm monomeric or aggregated P301S Tau, followed by 3 days of incubation. A GAPDH loading control is also shown.

Both monomeric and aggregated recombinant full-length Tau were internalized by cells at 37 °C, with similar kinetics over 120 min, suggesting a common uptake mechanism (Fig. 4*A*). Furthermore, the uptake of both Tau species over 30 min was greatly reduced at 4 °C and in the presence of 100 μm EIPA (Fig. 4*B*). A similar reduction by 300 nm latrunculin A was also observed (data not shown). This is consistent with endocytosis by macropinocytosis. Only aggregated Tau species were able to induce the aggregation of soluble Tau in the cytosol, giving rise to Sarkosyl-insoluble Tau composed of the expressed 1N Tau isoform. The latter failed to form in cells exposed to monomeric Tau (Fig. 4*C*).

##### Native Tau Aggregates Have a Higher Seeding Potency than Recombinant Tau Aggregates

**FIGURE 5.**
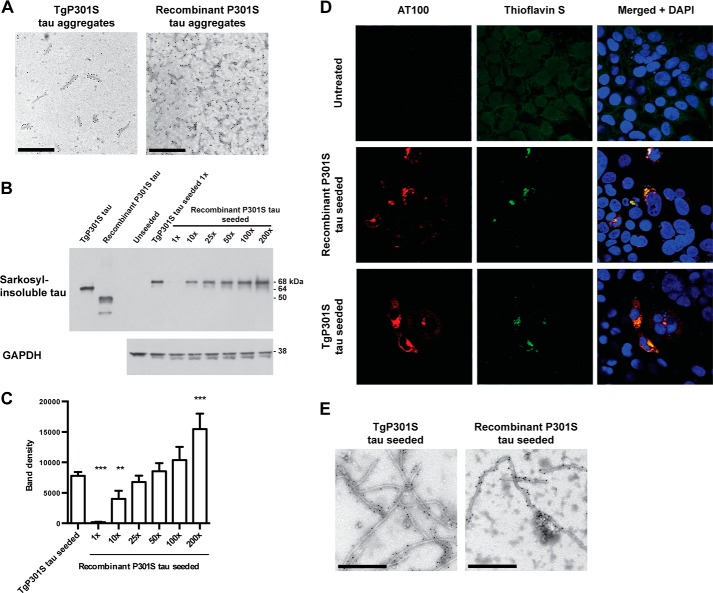
*A*, immunoelectron microscopy with anti-human Tau antibody HT7 (phosphorylation-independent) of the Sarkosyl-insoluble fraction from the brains of symptomatic TgP301S Tau mice and from recombinant P301S Tau assembled with heparin. Recombinant P301S Tau aggregates are shown following a 15-s sonication (Misonix, output 7). *Scale bars*, 400 nm. *B*, representative immunoblot with HT7 and quantification of the Sarkosyl-insoluble fraction of HEK 293T cells expressing 1N4R P301S Tau treated for 3 h with TgP301S Tau aggregates or with varying concentrations of recombinant Tau aggregates, followed by 3 days of incubation. At equal concentration (1×; equivalent to 7.2 nm, assuming complete assembly) recombinant P301S Tau did not seed. Over 10-fold higher concentrations of recombinant P301S Tau than TgP301S Tau aggregates were needed to give equivalent levels of seeding. The results are the means ± S.D. (*error bars*) (*n* = 3); **, *p* < 0.01; ***, *p* < 0.001 *versus* TgP301S Tau seeded (analysis of variance). *C*, immunofluorescence showing AT100 (Ser(P)^212^/Thr(P)^214^/Thr(P)^217^)-positive Tau inclusions and thioflavin S staining of HEK 293T cells expressing P301S 1N4R Tau, exposed to 1× TgP301S Tau aggregates or 50× recombinant P301S Tau aggregates. Nuclei were visualized by DAPI (*blue*). *D*, immunoelectron microscopy with anti-1N Tau antibody (phosphorylation-independent) of the Sarkosyl-insoluble fraction from HEK 293T cells expressing P301S 1N4R Tau treated for 3 h with 1× TgP301S Tau aggregates or 50× recombinant P301S Tau aggregates, followed by 3 days of incubation.

We compared Sarkosyl-insoluble Tau aggregates from the brains of TgP301S Tau mice with heparin-assembled full-length recombinant P301S 0N4R Tau. Immunoelectron microscopy with anti-Tau antibody HT7 showed that TgP301S human Tau existed as short, straight filaments with a mean length of 203 ± 83 nm (*n* = 50). Assembled recombinant P301S Tau comprised filaments with a mean length of 578 ± 363 nm (*n* = 50). To equate filament lengths, we sonicated the recombinant Tau filament preparation for 15 s (Misonix XL, output 7) to produce filaments with a mean length of 179 ± 82 nm (*n* = 50) (Fig. 5*A*). Both preparations bound thioflavin T (data not shown).

We normalized the concentration of TgP301S Tau aggregates to the sonicated recombinant P301S Tau aggregates by immunoblotting and assessed their seeding potencies in the cell-based assay (Fig. 5, *B* and *C*). Only the TgP301S Tau aggregates induced the formation of Sarkosyl-insoluble, hyperphosphorylated Tau from expressed protein. However, with increasing concentrations of recombinant P301S Tau aggregates, Sarkosyl-insoluble, hyperphosphorylated Tau formed from expressed protein, as seen by immunoblotting with HT7 (phosphorylation-independent) (Fig. 5*B*) and AT100 (Ser(P)^212^/Thr(P)^214^/Thr(P)^217^) and labeling with thioflavin S (Fig. 5*C*). Approximately 50 times more synthetic P301S Tau seed than TgP301S Tau seed was needed to produce equivalent amounts of Sarkosyl-insoluble Tau from expressed protein (Fig. 5, *B* and *C*). Immunoelectron microscopy of the Sarkosyl-insoluble fraction from cells seeded with either TgP301S Tau aggregates or recombinant P301S Tau aggregates showed the presence of Tau filaments made of expressed 1N Tau (Fig. 5*D*). This indicates that, although both native and recombinant P301S Tau aggregates can seed the formation of Sarkosyl-insoluble, hyperphosphorylated Tau in cells expressing soluble P301S Tau, native Tau aggregates are more potent.

##### Native Tau Aggregates Enter Cells through the Same Mechanism as Recombinant Tau Aggregates

**FIGURE 6.**
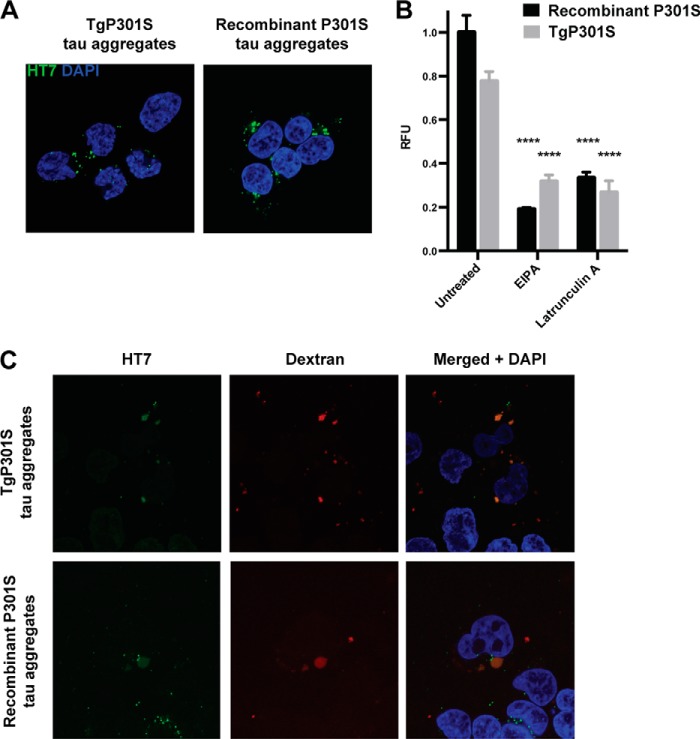
*A*, confocal imaging of HEK293T cells exposed to 500 nm TgP301S Tau aggregates or 500 nm recombinant P301S Tau aggregates for 3 h, followed by immunostaining with anti-Tau antibody HT7 (phosphorylation-independent). Punctate, often perinuclear, inclusions of internalized Tau aggregates can be distinguished. *B*, uptake of 500 nm fluorescently labeled immunopurified TgP301S Tau aggregates and recombinant P301S Tau aggregates after 30 min measured by flow cytometry. Incubation with 100 μm EIPA or 300 nm latrunculin A significantly inhibited the uptake of both TgP301S Tau and recombinant Tau aggregates. The results are the means ± S.D. (*error bars*) (*n* = 3); ****, *p* < 0.0001 *versus* untreated. *RFU*, relative fluorescence units; 10,000 cells/well were analyzed by flow cytometry. *C*, confocal imaging of HEK293T cells exposed to 50 μg/ml fluorescent dextran plus 500 nm TgP301S Tau aggregates or recombinant P301S Tau aggregates for 1 h, followed by immunostaining with HT7. Internalized Tau aggregates co-localized with dextran in large vesicles resembling macropinosomes.

We wanted to exclude the possibility that the difference in seeding between TgP301S Tau aggregates and recombinant P301S Tau aggregates was the result of the two preparations entering cells through different mechanisms. Both 500 nm TgP301S Tau aggregates and 500 nm recombinant Tau aggregates were taken up by cells within 3 h, as detected by immunohistochemistry (Fig. 6*A*). In order to quantify uptake, we immunopurified TgP301S Tau aggregates and confirmed that this material had characteristics similar to those of Sarkosyl-extracted TgP301S Tau (data not shown). This allowed us to directly label the Tau aggregates with DyLight 488 and to remove potential contaminating factors. We then applied equal concentrations of TgP301S Tau aggregates and recombinant P301S Tau aggregates to cells for 30 min and assessed uptake by measuring the mean fluorescence intensity of cells by flow cytometry (Fig. 6*B*, untreated). The mean fluorescence intensity of TgP301S Tau aggregate-treated cells was 77.6 ± 4.4% that of the recombinant P301S Tau aggregate-treated cells despite the TgP301S Tau aggregates displaying a significantly greater seeding potency (Fig. 5, *B* and *C*). We therefore concluded that the different seeding potencies of TgP301S Tau aggregates and recombinant P301S Tau aggregates were not due to differences in uptake efficiency.

Uptake of both TgP301S Tau aggregates and recombinant P301S Tau aggregates was significantly reduced by treating the cells with EIPA (100 μm) or latrunculin A (300 nm) for 30 min prior to and during the 30-min exposure to Tau aggregates, suggesting a shared uptake mechanism consistent with macropinocytosis (Fig. 6*B*). Treatment with EIPA reduced the mean fluorescence intensity of TgP301S Tau aggregate-treated cells by 59.2 ± 3.9% and recombinant P301S Tau aggregate-treated cells by 80.9 ± 0.7%. Similarly, treatment with latrunculin A reduced the mean fluorescence intensity of TgP301S Tau aggregate-treated cells by 65.6 ± 6.8% and recombinant P301S Tau aggregate-treated cells by 66.7 ± 2.7%. We conclude that the different seeding potencies of TgP301S Tau aggregates and recombinant P301S Tau aggregates were not caused by different uptake mechanisms. We also observed co-localization of TgP301S Tau aggregates and recombinant P301S Tau aggregates with the fluid phase endocytosis cargo dextran (Fig. 6*C*).

##### Recombinant Tau Seeded with Native Tau Aggregates Acquires the Same Seeding Potency as Native Tau Aggregates

**FIGURE 7.**
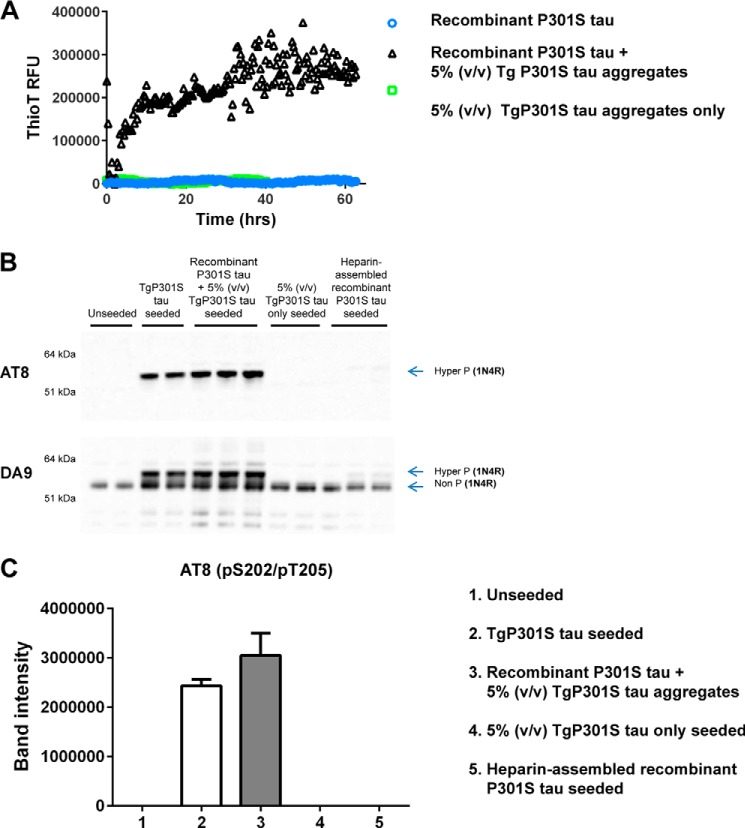
*A*, *black triangles* show the aggregation of soluble recombinant P301S Tau in the presence of 5% (v/v) Sarkosyl-insoluble fraction derived from the brains of mice transgenic for human mutant P301S Tau, as measured by thioflavin T binding over time. Heparin was not used. *Blue circles* and *green squares* show the thioflavin T traces for soluble recombinant P301S Tau and 5% (v/v) TgP301S Tau aggregates, respectively. *RFU*, relative fluorescence units. *B*, Western blots with anti-Tau antibodies DA9 (phosphorylation-independent) and AT8 (Ser(P)^202^/Thr(P)^205^) of the insoluble fraction from inducible HEK293T cells expressing P301S 1N4R Tau seeded for 3 h with TgP301S Tau aggregates (at a concentration equivalent to Fig. 5*B*, 1×); equivalent concentration of recombinant P301S Tau seeded with 5% (v/v) TgP301S Tau aggregates; equivalent concentration of heparin-assembled recombinant P301S Tau aggregates; or the 5% (v/v) TgP301S Tau aggregate component alone followed by 3 days of incubation. *C*, quantification of the AT8-positive insoluble fraction from *B*. The values are the means ± S.D. (*error bars*) (*n* = 3). Seeding with TgP301S Tau aggregates and recombinant P301S Tau seeded with (5%, v/v) TgP301S Tau aggregates were not significantly different.

To examine the effects of aggregate conformation on the potency of Tau seeding and to control for the presence of heterologous components in the TgP301S aggregates, we seeded recombinant P301S Tau in the absence of heparin with 5% (v/v) Sarkosyl-insoluble fraction from the brains of mice transgenic for human mutant P301S Tau. Recombinant P301S Tau aggregated, as measured by thioflavin T binding over time (Fig. 7*A*). Equivalent amounts of recombinant P301S Tau that had aggregated in the presence of heparin failed to induce the aggregation of recombinant P301S Tau (data not shown). When normalized for total Tau, the recombinant Tau aggregates seeded by aggregated TgP301S Tau in the absence of heparin were equally potent as aggregated TgP301S Tau in seeding assays (Fig. 7, *B* and *C*).

##### Hyperphosphorylation Does Not Increase the Seeding Potency of Recombinant Tau Aggregates

**FIGURE 8.**
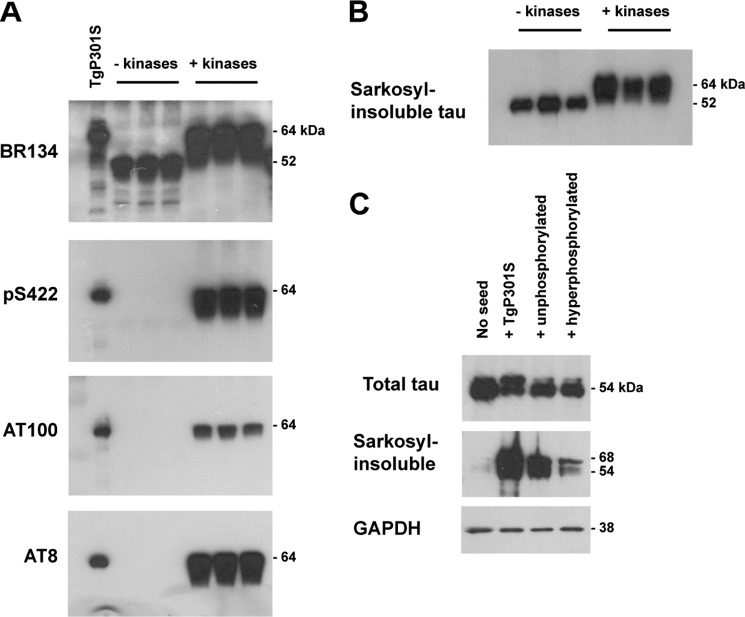
*A*, Western blot with anti-Tau antibodies BR134 (phosphorylation-independent; *top panel*), Ser(P)^422^ (*panel 2*), AT100 (Ser(P)^212^/Thr(P)^214^/Thr(P)^217^; *panel 3*), and AT8 (Ser(P)^202^/Thr(P)^205^; *panel 4*) of 0N4R P301S recombinant Tau treated with (+ kinases) or without (− kinases) PKA and SAPK4. Sarkosyl-insoluble TgP301S Tau is shown as a control. *B*, Western blot with anti-Tau antibody HT7 (phosphorylation-independent) of Sarkosyl-insoluble recombinant P301S Tau following treatment with (+ *kinases*) or without (− *kinases*) PKA and SAPK4 followed by assembly with heparin. *C*, Western blot with anti-Tau antibody HT7 of the total lysate (*top*) and Sarkosyl-insoluble fraction (*middle*) from unseeded cells (*no seed*) or cells seeded for 3 h with either TgP301S Tau aggregates (+ *TgP301S*), unphosphorylated recombinant P301S Tau aggregates (+ *unphosphorylated*), or hyperphosphorylated recombinant P301S Tau aggregates (+ *hyperphosphorylated*), followed by 3 days of incubation. GAPDH loading control is shown (*bottom*).

TgP301S Tau aggregates are hyperphosphorylated, whereas recombinant P301S Tau aggregates are unphosphorylated. To test whether this could account for the increased seeding potency of TgP301S Tau, we produced hyperphosphorylated recombinant P301S Tau aggregates *in vitro*. Sequential phosphorylation of recombinant P301S Tau with PKA and SAPK4, in the presence of heparin, gave rise to 64-kDa hyperphosphorylated Tau, as described previously (50). Notably, phosphorylation was detected at Ser^422^ (Fig. 8*A*, *panel 2*), the AT8 epitope (Ser(P)^202^/Thr(P)^205^; Fig. 8*A*, *panel 4*), and the AT100 epitope (Ser(P)^212^/Thr(P)^214^/Thr(P)^217^; Fig. 8*A*, *panel 3*). Hyperphosphorylation of these sites is characteristic of pathological Tau (Fig. 8*A*, *lane 1*) (59).

Hyperphosphorylation did not influence the assembly of recombinant P301S Tau with heparin, and we confirmed that hyperphosphorylation was retained following purification of Sarkosyl-insoluble Tau (Fig. 8*B*) (50).

We compared the seeding abilities of hyperphosphorylated and unphosphorylated recombinant P301S Tau aggregates as well as of 10-fold diluted TgP301S Tau aggregates (Fig. 8*C*). As described previously, TgP301S Tau aggregates were more potent seeds than unphosphorylated recombinant P301S Tau aggregates, even at a 10-fold dilution. Hyperphosphorylated recombinant P301S Tau aggregates were no more potent than unphosphorylated recombinant P301S Tau aggregates (Fig. 8*C*, *middle panel*).

##### Conformation Determines Seeding Potency of Tau Aggregates

**FIGURE 9.**
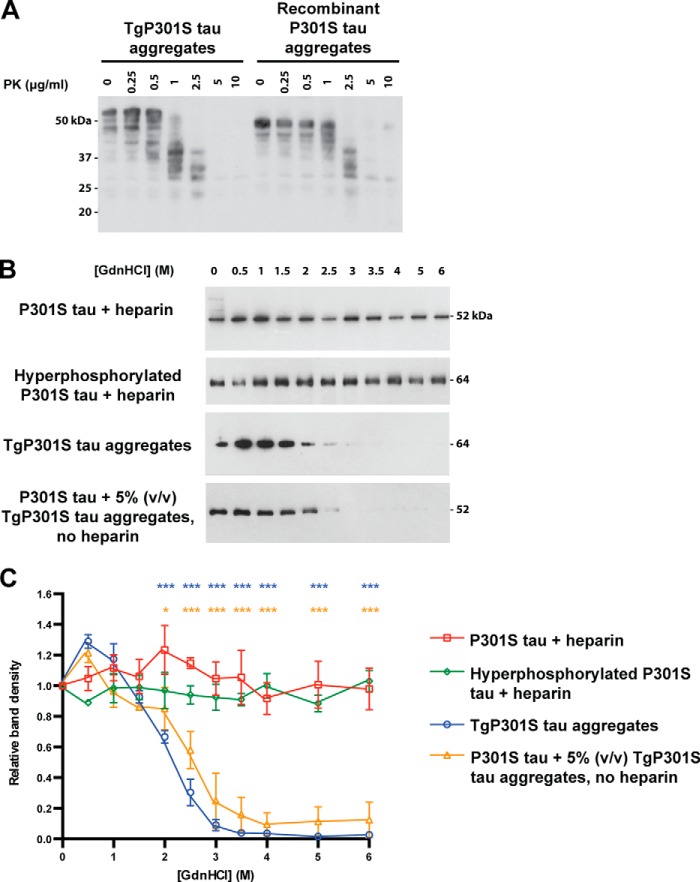
*A*, Western blot with anti-Tau antibody BR135 (phosphorylation-independent) of TgP301S Tau aggregates and heparin-assembled recombinant P301S Tau aggregates after digestion with increasing concentrations of proteinase K (*PK*). Recombinant P301S Tau aggregates were more resistant to proteinase K digestion than TgP301S Tau aggregates. *B* and *C*, Western blots with anti-Tau antibody HT7 (phosphorylation-independent) of heparin-assembled recombinant P301S Tau aggregates (P301S Tau + heparin; *red squares*); hyperphosphorylated, heparin-assembled recombinant P301S Tau aggregates (hyperphosphorylated P301S Tau + heparin; *green diamonds*); TgP301S Tau aggregates (*blue circles*); and recombinant P301S Tau seeded with 5% (v/v) TgP301S Tau aggregates (P301S Tau + 5% (v/v) TgP301S Tau aggregates, no heparin; *orange triangles*) following treatment with increasing concentrations of guanidine hydrochloride (*GdnHCl*) and centrifugation at 100,000 × *g* to pellet remaining Tau aggregates. Unphosphorylated and hyperphosphorylated recombinant P301S Tau aggregates were resistant to treatment with up to 6 m guanidine hydrochloride; TgP301S Tau aggregates and recombinant P301S Tau seeded with TgP301S Tau aggregates were partially solubilized with as little as 2 m guanidine hydrochloride, and their solubilization increased in a concentration-dependent manner. *C*, quantification of *B*. The results are the means ± S.D. (*error bars*) (*n* = 3; *, *p* < 0.05; ***, *p* < 0.001 *versus* P301S Tau + heparin (analysis of variance)).

To further test the hypothesis that conformation determines the seeding potency of Tau aggregates, we examined the effect of aggregate digestion by proteinase K using immunoblotting with anti-Tau antibody BR135 (repeat region) (Fig. 9*A*). Recombinant P301S Tau aggregates were more resistant to proteinase K digestion than TgP301S Tau aggregates.

We then used a guanidine stability assay to further examine conformational differences between TgP301S Tau aggregates and recombinant Tau aggregates. We also evaluated recombinant Tau seeded by TgP301S Tau aggregates in the absence of heparin and hyperphosphorylated recombinant Tau aggregates assembled in the presence of heparin (Fig. 9, *B* and *C*). Recombinant P301S Tau aggregates were significantly more resistant to disaggregation by guanidine hydrochloride than TgP301S Tau aggregates. Moreover, treatment with low concentrations of guanidine hydrochloride increased the detection of TgP301S Tau aggregates by HT7 (phosphorylation-independent), probably through increased exposure of its epitope. Similar results were also obtained with DA9, another phosphorylation-independent anti-Tau antibody (data not shown). This was not observed for recombinant P301S Tau aggregates. Recombinant Tau seeded by TgP301S Tau aggregates in the absence of heparin displayed stability to guanidine hydrochloride similar to that of TgP301S Tau aggregates; it was significantly different from the guanidine stability of heparin-assembled recombinant Tau aggregates. Hyperphosphorylated heparin-assembled recombinant Tau aggregates did not show guanidine hydrochloride stability significantly different from that of unphosphorylated heparin-assembled recombinant Tau aggregates.

These findings suggest that there are significant conformational differences between native and heparin-assembled recombinant Tau aggregates, which are reflected in their respective seeding potencies. Moreover, unphosphorylated recombinant Tau can acquire the conformation of TgP301S Tau through *in vitro* seeding.

##### Aggregation of Expressed Tau Is Required for Seeding

**FIGURE 10.**
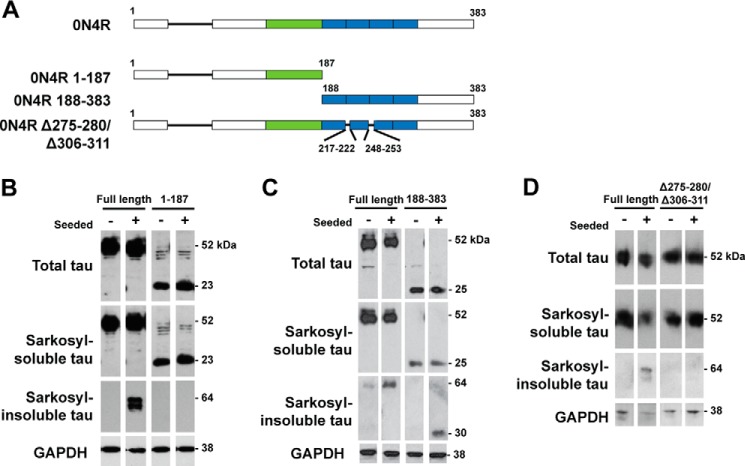
*A*, schematic diagram of the 0N4R Tau constructs used. *B*, Western blot with anti-Tau antibody BR133 (phosphorylation-independent) of HEK293T cells expressing full-length 0N4R Tau or 0N4R Tau 1–187, which were seeded for 3 h with TgP301S Tau aggregates, followed by 3 days of incubation. *C*, Western blot with anti-Tau antibody BR134 (phosphorylation-independent) of HEK293T cells expressing full-length 0N4R Tau or 0N4R Tau 188–383, which were seeded for 3 h with TgP301S Tau aggregates followed by 3 days of incubation. *D*, Western blot with BR134 of HEK293T cells expressing either 0N4R Tau or 0N4R Tau ΔPHF6/6*, which were seeded for 3 h with TgP301S Tau aggregates, followed by 3 days of incubation.

We investigated the regions of expressed soluble full-length Tau required for seeding (Fig. 10*A*). HEK 293T cells expressing 0N4R Tau were exposed to the Sarkosyl-insoluble fraction from the brains of symptomatic TgP301S Tau mice for 3 h, followed by 3 days of growth. Seeding was evaluated by the detection of Sarkosyl-insoluble, hyperphosphorylated Tau. 0N4R Tau lacking the repeat region (amino acids 1–187) was incapable of being seeded (Fig. 10*B*), whereas its carboxyl-terminal half with the repeats (amino acids 188–383) could be seeded (Fig. 10*C*). Cells expressing full-length 0N4R Tau lacking residues ^275^VQIINK^280^ and ^306^VQIVYK^311^ (Δ275–280/Δ306–311), could not be seeded (Fig. 10*D*).

## DISCUSSION

Using a cell-based assay, we compared the seeding activity of the Sarkosyl-insoluble fraction from the brains of symptomatic TgP301S Tau mice with that of total brain lysate. The Sarkosyl-insoluble fraction, which contained filamentous Tau, had a much greater seeding activity than the lysate, when normalized to total Tau. This indicates that the Tau species required for seeding are likely to be Sarkosyl-insoluble. Monomeric Tau was devoid of seeding activity, as was Sarkosyl-soluble Tau lacking aggregated Tau species (data not shown), showing the need for aggregation. Aggregation was also necessary for the ability of Tau to undergo seeding because Tau proteins incapable of aggregation could not be seeded. These findings using full-length Tau are in agreement with work showing that the seeding of tagged Tau repeats was aggregation-dependent (60). In the human tauopathies, full-length Tau makes up the abnormal filaments (1).

The Sarkosyl-insoluble fraction from TgP301S Tau mouse brain was enriched in aggregated and AT8-positive Tau. However, there are a number of potential Tau species, including small oligomers, which may contribute to the seeding activity. Further studies are required to characterize the full range of seed-competent species in the TgP301S Tau model.

These findings are reminiscent of the behavior of prions consisting of conformationally modified prion protein (61, 62) and indicate that assembled Tau protein exhibits prion-like properties. The production of synthetic prions capable of propagation has been difficult, and they display long incubation times compared with their native counterparts (63). The same has been observed for synthetic assembled Aβ in transgenic mice (64, 65). Moreover, mice injected with a small amount of native aggregated α-synuclein propagated pathology with a potency similar to that of mice injected with a large amount of synthetic α-synuclein aggregates (66). Similarly, in yeast, an inverse correlation between prion proliferation and stability has been described (67). In prions, there is a negative correlation between structural stability and seeding activity (68, 69). The fact that synthetic prions are generally more stable than native prions offers an explanation for their reduced seeding potential.

Here we show that, similar to synthetic prions, α-synuclein aggregates, and Aβ assemblies, synthetic Tau aggregates had a greatly reduced seeding activity compared with Sarkosyl-insoluble Tau from TgP301S Tau mouse brain. It has been suggested that the lower potency of synthetic prions, α-synuclein aggregates, and assembled Aβ could be the result of the presence of multiple molecular species, only some of which had seeding activity. We used sonication to create a homogeneous preparation of Sarkosyl-insoluble synthetic Tau filaments, as characterized by electron microscopy, reducing the likelihood of differences in filament length being the reason for the lower seeding ability. Synthetic Tau aggregates were more resistant to disaggregation by guanidine hydrochloride and digestion by proteinase K than Tau aggregates from TgP301S Tau mouse brain, consistent with the hypothesis that more stable aggregates possess lower seeding activity. These findings are in agreement with a study showing that Tau filaments produced by incubating recombinant human Tau with heparin were more stable than paired helical filaments from AD brain (70).

Native and synthetic Tau aggregates may have distinct conformations. We used heparin to form synthetic aggregates; it compacts the repeats and induces dimerization of Tau, with filaments growing through monomer addition (22, 23). This may give synthetic aggregates a conformation different from that of native Tau aggregates. Unlike synthetic aggregates, disaggregation of Tau aggregates from TgP301S Tau mouse brains with low concentrations of guanidine hydrochloride enhanced exposure of the HT7 epitope, suggesting a different molecular organization of the two types of aggregates. Moreover, the two types of aggregates exhibited different digestion patterns with proteinase K. Consistent with this, we produced synthetic Tau filaments seeded with TgP301S Tau aggregates that showed a resistance to disaggregation by guanidine hydrochloride similar to that of TgP301S Tau seeds. Importantly, synthetic Tau aggregates seeded by aggregated TgP301S Tau in the absence of heparin acquired the seeding potency of brain-derived TgP301S Tau seeds. This is consistent with the prion concept, which postulates that the conformational properties of the seed determine the type of aggregate formed (61). In mice, stable synthetic prions exhibited longer incubation times than their more labile counterparts (71).

We cannot rule out the possibility that post-translational modifications may reduce the stability of Tau aggregates because filaments from TgP301S Tau mouse brains were hyperphosphorylated (48) and may have carried additional modifications. Synthetic Tau aggregates, by contrast, are devoid of post-translational modifications. To test whether phosphorylation could account for the increased seeding potency of aggregated TgP301S Tau, we generated hyperphosphorylated recombinant P301S Tau aggregates *in vitro* and tested their seeding potency. Hyperphosphorylated recombinant P301S Tau aggregates were no more potent at seeding than unphosphorylated recombinant P301S Tau aggregates, demonstrating that phosphorylation was not necessary for seeding activity, although the seeded Tau aggregates were hyperphosphorylated and may have carried additional modifications. It is important to note that the recombinant Tau seeded with TgP301S Tau aggregates did not carry any post-translational modifications either but was able to seed as efficiently as TgP301S Tau aggregates. Moreover, seeding efficiency was not reduced in cell lines expressing Tau incapable of undergoing phosphorylation at disease-specific sites.^9^ Taken together, these findings support the conclusion that conformation, not hyperphosphorylation, drives the seeding potency of Tau aggregates. Further work is required to identify the role, if any, of additional phosphorylation and other post-translational modifications.

Macropinocytosis has been implicated in the uptake of aggregated Tau repeats into cells in culture (46). We found that both native and synthetic Tau aggregates entered cells through a mechanism consistent with macropinocytosis. This demonstrates that the difference in seeding activity between native and synthetic Tau aggregates is unlikely to be the result of different uptake mechanisms. We also found that both monomeric and aggregated Tau enter cells by a mechanism consistent with macropinocytosis with equal efficiency. This stands in contrast to monomeric and fibrillar α-synuclein, which has been reported to be taken up through different mechanisms (72).

Elucidation of the molecular mechanisms underlying aggregation and propagation is important for understanding the pathogenesis of the tauopathies, which appear to originate in defined brain regions, before spreading through the brain (26–29). This may also be relevant for other neurodegenerative diseases (51, 64, 66–68, 73–76).

Detailed characterization of the molecular species responsible for propagation is important for uncovering the mechanisms underlying seeding and spreading, for developing diagnostic tests, and for devising therapies that target these species (such as immunization, inhibition of fibril assembly, and enhanced cellular degradation).

The fact that the conformation of aggregated Tau can determine its seeding characteristics may provide a molecular explanation for the tauopathies, which are defined by the presence of Tau inclusions in the brain but have distinct neuropathologies and rates of progression. It has been shown that the intracerebral injection of brain homogenates from human 4R tauopathies (AGD, progressive supranuclear palsy, and corticobasal degeneration) into mice transgenic for one isoform of wild-type 4R human Tau seeded the formation of pathological inclusions like those seen in the human tauopathies (37). Future work will focus on the molecular differences between Tau aggregates from distinct tauopathies because we show here that conformation determines the seeding potencies of native and recombinant Tau aggregates.

## Footnotes

9 B. Falcon and M. Goedert, unpublished results unpublished results.

## Footnotes

8 The abbreviations used are:

AD: Alzheimer disease
AGD: argyrophilic grain disease
SAPK4: stress-activated protein kinase 4
EIPA: ethylisopropyl amiloride
bis-tris: 2-[bis(2-hydroxyethyl)amino]-2-(hydroxymethyl)propane-1,3-diol.
